# Microbiota—A Rescuing Modulator in Children Struggling with Functional Constipation

**DOI:** 10.3390/microorganisms13071504

**Published:** 2025-06-27

**Authors:** Nicoleta Ana Tomșa, Lorena Elena Meliț, Teodora Popescu, Karina Najjar, Anca Meda Văsieșiu, Adrian Vlad Pop, Reka Borka-Balas

**Affiliations:** 1 Doctoral School of Medicine and Pharmacy, “George Emil Palade” University of Medicine, Pharmacy, Science and Technology of Targu Mures, Gheorghe Marinescu Street, No. 38, 540136 Targu Mures, Romania; tomsa.nicoleta@yahoo.com (N.A.T.); teo7ro@yahoo.com (T.P.); 2 Department of Pediatrics II, “George Emil Palade” University of Medicine, Pharmacy, Sciences and Technology of Targu Mures, Gheorghe Marinescu Street, No. 38, 540136 Targu Mures, Romania; karinanajjar792@gmail.com; 3 Department of Infectious Diseases, “George Emil Palade” University of Medicine, Pharmacy, Sciences and Technology of Targu Mures, Gheorghe Marinescu Street, No. 38, 540136 Targu Mures, Romania; anca-meda.vasiesiu@umfst.ro (A.M.V.);; 4 Department of Pediatrics I, “George Emil Palade” University of Medicine, Pharmacy, Sciences and Technology of Targu Mures, Gheorghe Marinescu Street, No. 38, 540136 Targu Mures, Romania; rekaborkabalas@gmail.com

**Keywords:** constipation, children, probiotics

## Abstract

Constipation affects around 30% of children and in 95% of cases is functional (FC), a consequence of alterations in digestive tract peristalsis, modulated by the immune and nervous systems, bile acid metabolism, and the gut microbiota. The aim of this review was to assess the role of gut microbiota and the use of probiotics in children with constipation. The current treatment involves education, toilet training, and oral laxatives, effective in only 50% of patients. In chronic FC, the composition of the microbiota is altered, with increased abundance of *Bacteroidetes*, *Enterobacteriaceae*, and *Firmicutes* and decreases in *Prevotella*, *Bifidobacteria*, *Faecalibacterium prausnitzii*, and *Clostridium leptum*. Probiotics replenish lacking beneficial resident bacteria, downregulate mucosal inflammation, or produce short-chain fatty acids (SCFAs). Probiotics like *Bifidobacterium breve* and *Bifidobacterium longum* increase the defecation frequency and decrease the episodes of both fecal incontinence and abdominal pain. *Bifidobacterium animalis* subsp*. lactis XLTG11* improves the gut microbiota by upregulating SCFA genes and downregulating those related to methane metabolism. *Lactobacilli* produce organic acids that stimulate bowel peristalsis and augment fecal bolus moisture. The heterogeneity of the current studies involving pediatric subjects thus far hinders the use of probiotics as a standard in the management of children with constipation.

## 1. Introduction

Constipation is a common worldwide problem affecting all ages, which is usually defined as difficult and infrequent elimination of increased consistency, with a small amount of stools, i.e., under three bowel movements per week [1]. The prevalence of this condition presents a continuous increase, lately varying between 2.5% and 79% in adults, and between 0.7% and 29.6% in children [2]. More recent evidence indicates higher prevalence rates of up to 32.2%, with toddlers being the most affected age group [3]. Moreover, according to a study performed in the period 1997–2009, in the USA, the number of children admitted for constipation increased by 112%, and its associated costs increased by 221.5% [4]. Constipation in children is diagnosed as functional in approximately 95% of cases, with only 5% of the patients being identified as having an organic origin of this condition [5].

Unfortunately, the precise etiology of functional constipation (FC) remains uncertain, but conscious retention of defecation because of fear or pain is considered to be the main causal agent causing the well-known ‘vicious circle’ in constipation [4,6,7,8]. This type of retentional behavior is considered to be the leading cause of constipation in younger children [4]. The consequence of stool withholding consists in long-term feces stasis, with their subsequent accumulation in the rectum and its enlargement, which will eventually lead to a deficit in anal muscle contraction, impairment of pelvic floor muscle function, and a major impairment of anal sphincter function [4,7]. All of the aforementioned events contribute to the occurrence of fecal incontinence in almost 30% of pediatric patients with FC [8]. Furthermore, FC is classified into normal- and slow-transit constipation, as well as rectal or defecatory evacuation disorders [9]. Other factors involved in the etiology of FC include bowel motility disorders, gut microbiota, pelvic floor spasms, decreased fiber intake, lack of physical activity, obesity, poor socioeconomic status, low maternal education, stress, insufficient sleep or attention from parents, and genetic factors [5].

The Rome IV criteria are the most commonly used to diagnose constipation in children. These criteria classify children with constipation into two categories, depending on their toilet coaching status: children with toilet coaching, and those lacking this habit. Thus, in children without toilet training, FC should be diagnosed if they fulfill at least two of the following criteria: a maximum of two eliminations in seven days associated with a history of stool retention, extremely hard-consistency stools, agonizing defecation, increased fecal width, and/or the identification of a large fecaloma stuck within the rectum. In terms of children with toilet training, the Rome experts included two supplementary criteria: a history of giant-sized stools that usually block the toilet, and/or at least one event of fecal incontinence every seven days [10,11,12].

The management of children with FC is definitely a struggle, due to a wide spectrum of factors related to the condition itself, the patient and caregivers, the doctor, and the treatment. In fact, the relationship among all of these factors frequently contributes to the poor long-term outcomes in these children, since the complex etiology of FC involves a complex treatment based especially on lifestyle changes and osmotic laxatives, leading subsequently to increased costs due its prolonged evolution and major economic, social, physical, and emotional negative consequences [5,13,14,15]. Moreover, the doctors’ knowledge gaps regarding FC and their refusal to use the standard Rome diagnostic criteria, as well as their reluctance to recommend non-pharmacological interventions in children suffering from FC, consistently impair the outcomes of these patients, creating major geographical discrepancies between the protocol and clinical practice [16].

## 2. Materials and Methods

We electronically searched articles on PubMed^®^ by including the following key words: constipation, probiotics, synbiotics, microbiota, fecal microbiota transplantation, and children; the key word children was associated with each of the other keywords during the search. We initially included all articles referring to children with constipation and the assessment of microbiota and probiotics in these children. Each of these publications was critically assessed according to the following: key results, quality of the results, interpretation of the results, suitability of the methods used to verify the hypothesis, limitations, and impact of the conclusions to the field [17]. Thus, all articles that did not meet these criteria, along with those for which the full text was not available, were excluded.

**Figure 1:**
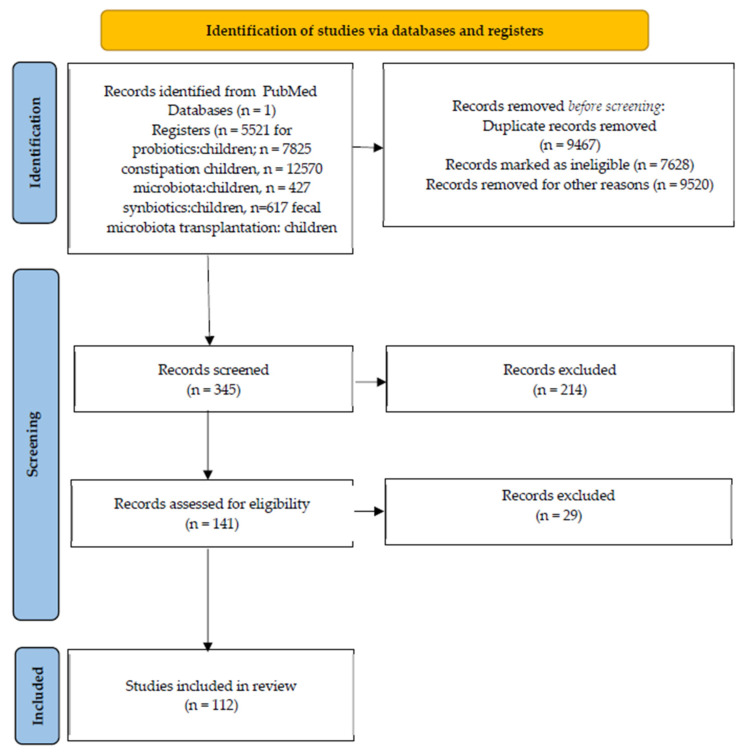
Search methodology.

We also found additional references of interest for our topic by a manual search in the reference lists of the articles retrieved in the first round of search, and we followed the exact same steps as for the initial list of references before including them in the final reference list. All of the information related to the number of references, as well as the search methodology described above, can be found in Figure 1.

We synthesized all of the information found in the references in order to provide an objective narrative overview related to the impact of microbiota and probiotics in children with constipation.

This review required no ethical approval.

## 3. Microbiota Explains Impaired Intestinal Motility

Studies proved a strong relationship between chronic constipation and microbiota alterations, regardless of age [18,19]. In fact, the results underlined a reciprocal influence, since intestinal bacteria are responsible for 50% of the feces volume, while prolonged stasis of stools in the colon of individuals with constipation may alter the microbiota composition. Most of the evidence related to chronic constipation and microbiota composition so far suggests an increased abundance of *Bacteroidetes* and *Enterobacteriaceae*, including patients with irritable bowel syndrome manifesting predominantly constipation, along with a decrease in the genera *Bifidobacterium* and *Lactobacillus* [19,20,21]. Constipation is mainly a consequence of alterations in digestive tract peristalsis, but the normal function of the bowel is modulated by factors like the immune system, the nervous system, bile acid metabolism, and the resident microbiota of the digestive tract, together with its alterations [5]. A more thorough approach to this issue hypothesized that the bacterial endotoxin lipopolysaccharide, synthetized by Gram-negative bacteria like *Enterobacteria*, is able to delay gastric emptying and to induce sphincter dysfunction, leading eventually to decreased bowel movements [15]. Other studies indicated different changes in patients suffering from chronic constipation, such as higher abundance of *Firmicutes* and lower abundance of *Prevotella*; decreased representation of *Bifidobacteria, Faecalibacterium prausnitzii*, and *Clostridium leptum* in patients with irritable bowel syndrome and constipation; and an abundance of *Methanobrevibacter* strains, explaining the greater-than-normal production of methane in chronic constipation [6,20,21,22,23,24,25,26].

Although the available data are somehow contradictory, the causal relationship between gut microbial composition of resident bacteria and constipation was further proved by studies on gnotobiotic animals colonized with constipation patients’ fecal microbiota, expressing a reduction in intestinal peristalsis and abnormal defecation parameters [27]. Moreover, studies on animal models also showed that resident bacteria are involved in the development and maintenance of several gut functions, such as motor, sensory, vascular, and immune functions, resulting in delayed gastric emptying and increased gastrointestinal time as compared to conventionally raised animals [28]. In fact, recent studies have hypothesized several mechanisms that might be involved in the modulation of bowel physiology and motility. Aside from the aforementioned bacterial endotoxin lipopolysaccharide produced by Gram-negative bacteria living in the human gut, another study performed on germ-free rats that used the re-colonization of the intestinal tract proved that *Bifidobacteria* and *Lactobacillus* spp. might augment the release of serotonin, with pro-motility effects, by reducing the migrating myoelectric complex period and the subsequent acceleration of small gut transit [29]. Contrariwise, the same study pointed out that *Micrococcus luteus* and *Escherichia coli* strains had an opposite effect, showing an inhibitory effect on bowel transit [29]. Another important mechanism mentioned in animal studies was centered on the effect of short-chain fatty acids (SCFAs), including acetate, butyrate, and propionate, produced by certain strains residing in the gut microbiota in anaerobic conditions during fermentation. Thus, bacteria like *Clostridia, Bifidobacteria*, and *Faecalibacterium prausnitzii*, through their products, have the ability to stimulate the enteric cholinergic reflex by stimulating the release of GLP-1 or polypeptide YY from gut mucosal cells [30,31]. Furthermore, SCFAs eventually contribute to the acceleration of bowel transit due to serotonin release from enterochromaffin cells via vagal sensory fibers’ promotion [32]. In terms of butyrate, studies proved that it might express a dichotomous role, reducing or increasing bowel transit depending on its concentrations; thus, its positive effects are expressed only at increased concentrations, while at normal concentrations, butyrate induces constipation, since it promotes colonic electrolyte and water absorption and, at the same time, lowers colonic mucin secretion [33,34,35]. Resident gut microbes also express a metabolic function on bile acids secreting enzymes that hydrolyze these acids into non-reabsorbable secondary bile acids, which bind to their receptor, forming the G protein-coupled bile acid receptor, and enable enterochromaffin cells and intrinsic primary afferent neurons to release serotonin and calcitonin gene-related peptide, eventually acting as a trigger for the bowel’s peristaltic reflex [36,37]. Less studied mechanisms, such as the promotion of γ-aminoacidobutiric acid, enteric γ-actin, vesicle-associated protein-3, and the impairment of the colonic mucus layer, were also suggested to be involved in gut motility modulation [1]. All of the aforementioned information is synthesized in Table 1.

## 4. Probiotics and Constipation—Why, What, and When?

According to the World Health Organization and the Food and Agriculture Organization of the United Nations, probiotics are live microorganisms with a benefit on the host’s health if administered in proper amounts [38]. The positive impact of probiotics on the gut ecosystem of patients suffering from constipation has been explained by mechanisms like replenishing lacking beneficial resident bacteria, downregulating mucosal inflammation, or producing SCFAs [1,31,39,40]. Several mechanisms are emphasized regarding the reasons why probiotics might express a positive effect in constipation. One of them, and probably the most important, focuses on the fact that constipation is associated with dysbiosis, and that probiotics improve the composition of the gut microbiota, resulting in the improvement of constipation-related symptoms, although is it not completely understood whether dysbiosis precedes constipation or if it is a consequence of constipation [41,42,43]. Another mechanism is related to the colonic pH, since it is well documented that a lower pH is associated with better colonic peristalsis and subsequent faster colonic transit time. Studies prove that probiotics increase the production of short-chain fatty acids, especially lactic and acetic acids, resulting in a lower colonic pH [41,42].

Unfortunately, the scarcity of studies performed on pediatric subjects hinders the clarity of this topic regarding probiotics and constipation. Nevertheless, the available evidence sheds light on both changes in the gut microbiota in children with constipation and the effects of probiotics in treating these patients. The studies on probiotics and constipation were strongly motivated by the poor efficacy of current treatment methods in patients complaining of constipation. The current treatment used in this group of patients consists of education strategies, toilet training, and oral laxatives, but only half of the patients receiving oral laxatives for up to 12 months recovered completely when this medication was withdrawn [44]. Additionally, 25% of children who were diagnosed with constipation below the age of 5 years continued to experience symptoms of constipation during puberty despite aggressive medical and educational interventions [45]. Also, a major disadvantage of oral laxatives consists of the important side effects reported by these patients, such as bloating, abdominal pain, diarrhea, bad taste, or nausea [46].

**Figure 2:**
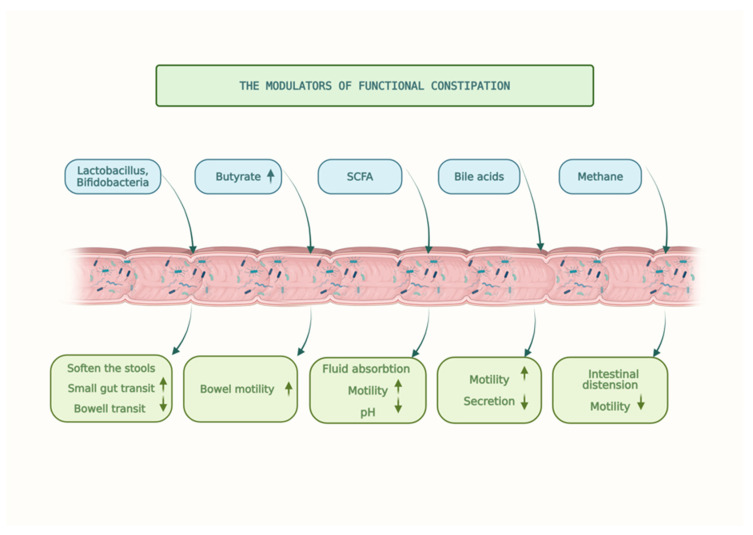
The modulators of functional constipation. (Legend: 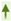 increase, 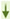 decrease).

The mechanisms involved in the treatment and prevention of functional constipation is described in Figure 2.

### 4.1. Bifidobacterium spp. in Children with Constipation

Studies on adult patients with constipation revealed that the consumption of Bifidus yoghurt containing *Bifidobacterium bifidum*, *Bifidobacterium breve*, and *Lactobacillus acidophilus* led to an increase in defecation frequency and had no side effects [47]. The positive impact of *Bifidobacterium breve* on gastrointestinal motility was highlighted more than 25 years ago by a study performed on preterm infants, where its administration was associated with less vomiting, less gas accumulation within the stomach, and improved weight gain, with no side effects [48]. In fact, the improper gut colonization of neonates was hypothesized to be involved in the development of certain functional gastrointestinal disorders [49,50]. Studies have highlighted that the gut microbiota of infants presenting colic is less diverse in content, harboring higher contents of Gram-negative bacteria and lower levels of *Lactobacillus* and *Bifidobacteria* [51,52,53].

Among the first studies performed on this topic in pediatric patients, in 1998, Zoppi et al. concluded after comparing the fecal microbiota of 28 children with constipation and 14 healthy controls (aged between 5 and 14 years) that children dealing with constipation harbor significantly greater abundances of the genera *Bifidobacteria* and *Clostridia* [54]. In terms of therapeutic efficacy, Tabbers et al. performed a study on 20 children with constipation who received *Bifidobacterium breve* for 4 weeks, and they proved that this probiotic increases the defecation frequency while significantly decreasing the episodes of both fecal incontinence and abdominal pain [43]. Moreover, the authors also noted that *Bifidobacterium breve* softened the stools, which, along with the decrease in abdominal pain episodes, might be due to the mechanisms of probiotics consisting in stimulating electrolyte and water secretion [55,56]. The positive effect of probiotics on abdominal pain was also proven in a study involving patients with irritable bowel syndrome who were administered *Bifidobacterium infantis*, with the authors hypothesizing that probiotics reduce hypersensitivity, expressing an anti-inflammatory impact on the enteric mucosa [57].

Makizaki et al. performed a study on rats in which constipation was induced by a low-fiber diet, followed by a combination of low-fiber diet and *Bifidobacterium bifidum G9-1* for 14 days, and proved that that this probiotic improves the dysbiosis caused by the low-fiber diet, leading to an increased defecation frequency, a higher content of water in the feces, and subsequently increased fecal weight [58]. Moreover, the authors pointed out that *Bifidobacterium bifidum G9-1* increases the concentration of organic acids at the cecum level. Another member of the *Bifidobacteria* genus is *Bifidobacterium longum*, which was studied by Guerra et al. in a study on 59 pediatric patients diagnosed with pediatric functional chronic intestinal constipation, proving that this probiotic significantly improves defecation frequency, alleviating defecation and abdominal pain as well [59]. A more recent study assessed the effect of *Bifidobacterium longum* associated with *Pediococcus pentosaceus* in infants with colic and functional constipation, proving that this combination of probiotics improved infants’ symptoms independent of their delivery (vaginal or C-section) or feeding pattern (breastfed, formula-fed, or both), as well as regardless of the other used treatments during probiotic administration [60]. In addition, the authors underlined that these two probiotic strains also possess a positive anti-infectious effect by antagonizing enteropathogens such as Gram-positive bacteria that are well known to cause pediatric infections [61]. This effect is related to the production of lactate and/or acetate organic acids, but most likely also to their potential ability to produce bacteriocin [60]. The findings of Chen et al. also indicate that the combination of these two probiotic strains is associated with shorter crying time and decreased crying episodes [62]. The same increase in defecation frequency was noted when using *Bifidobacterium animalis* subsp. *lactis XLTG11* for treating children with functional constipation, while also inducing positive changes in the gut microbiota, upregulating short-chain fatty acid-related genes, and downregulating those related to methane metabolism [63].

Similar findings regarding increased defecation frequency, fecal consistency improvement, and reductions in fecal incontinence episodes were also reported when using a mixture of probiotics containing *Bifidobacterium infantis*, *Bifidobacterium longum*, *Bifidobacterium bifidum*, *Lactobacillus plantarum*, *Lactobacillus rhamnosus*, and *Lactobacillus casei* [64].

Table 2 describes the most relevant information related to the role of *Bifidobacterium* spp. in children with constipation.

### 4.2. Lactobacillus spp. in Children with Constipation

Lactobacilli are another component of the gut microflora emphasized to have a positive impact in preventing constipation due to their ability to produce organic acids like lactic, acetic, or other acids, but also based on their potential to stimulate bowel peristalsis and to augment fecal bolus moisture [65]. It has been suggested that fermentation products like acetic acids have the ability to trigger smooth muscle contractions, depending on their concentration, and therefore to promote maximal contraction amplitudes [66]. According to culture-independent methods, it seems that the amount of autochthonous *Lactobacillus* represents approximately 1% of all bacterial residents in the human gut microbiota, with wide-spectrum variability among individuals in terms of certain *Lactobacillus spp.* [67]. Jomehzadeh et al. concluded that this amount of lactobacilli in the gut is insufficient for preventing functional constipation or other functional gastrointestinal disorders, since their quantity is essential for fulfilling their function [68]. The most frequently identified *Lactobacillus* spp. in the feces of healthy individuals are *Lactobacillus* (*L*.) *acidophilus*, *L. casei*, *L. reuteri*, *L. salivarius*, *L. rhamnosus*, *L. plantarum*, *L. brevis*, *L. fermentum*, and *L. paracasei* [69,70,71,72]. Jomehzadeh et al. sustained this hypothesis through their findings highlighting that *Lactobacillus* spp. are significantly decreased in quantity among children with functional constipation when compared to healthy controls [73]. Moreover, *L. reuteri* was defined as a modulator of gut motor behavior, since Wu et al. proved that it reduces the velocity and frequency of jejunal motor contractions, while it augments the same parameters of colonic motility [74]. It was also hypothesized that this probiotic is related to the gut neurons’ activity due to its potential for selectively increasing the excitability of neurons within the colon and inhibiting the tonic colonic contractile activity [75,76].

Among the few studies performed in children, a recent complex study assessed the role of *L. reuteri* DSM 17938 and agave inulin in pediatric patients diagnosed with cerebral palsy and constipation. The authors divided the sample into four groups, defined as follows: the probiotic group, who received *L. reuteri* and placebo; the prebiotic group, who received agave inulin with placebo; the synbiotic group, who were administered *L. reuteri* and agave inulin; and the placebo group, who were given two placebos for a period of 28 days. Although the sample size was relatively small, involving only thirty-seven patients, the authors noted that the stool pH decreased in the probiotic group, while the prebiotic group presented an improvement in stool consistency, and in all three groups that received *L. reuteri* and/or agave, the excessive stool retention and painful defecation episodes decreased [77]. Moreover, the authors showed that in children who received *L. reuteri*, its concentration increased by 60-fold in their feces, proving that this probiotic remains alive in the human gastrointestinal tract, and they reported that the increased concentration led to a significantly lower stool pH, an essential protective mechanism against pathogenic bacteria. The same strain of *L. reuteri*, i.e., DSM 17938, was assessed in Japanese children with constipation aged between 6 months and 6 years, who were sorted into three groups as follows: 20 children receiving *L. reuteri* and placebo (lactose hydrate), 19 children who were given *L. reuteri* and MgO, and 21 children treated with MgO and *L. reuteri*. The findings indicated that both *L. reuteri* DSM 17938 and MgO present positive effects on defecation frequency, but only MgO was associated with decreased stool consistency. Nevertheless, the study also concluded that MgO might induce negative changes in gut microbiota, inducing a reduction in the genus *Dialister* [78].

In terms of age, *L. reuteri* was also assessed in infants with constipation, and it was demonstrated to improve bowel movements and defecation frequency [79]. The underlying mechanism for constipation during infancy consists of the reduction in Bifidobacteria and Lactobacilli due to changes in diet such as the introduction of solid food or the passage from breastfeeding to milk formula, imbalances that are restored by *L. reuteri* [80]. Moreover, early life events also lead to major negative changes in the gut microbiome and subsequent changes in bowel movements, being also related to increased visceral sensitivity and mucosal permeability [80]. Thus, Indrio et al. highlighted the potential benefit of *L. reuteri* in reducing constipation in infants below the age of 3 months [81]. Another potential mechanism for the beneficial effect of *L. reuteri,* proven in adults with constipation, consists of its ability to act as a modulator of serum serotonin levels and brain-derived neurotrophic factor, resulting in reductions in abdominal pain or discomfort and bloating episodes, but also in increasing defecation frequency [82]. In addition, Dimidi et al., in their review on the role of probiotics in adults with constipation, underlined that *L. reuteri* regulates the ‘famous’ gut–brain axis and influences gut motility by modulating the afferent sensory nerves [65,83]. Nevertheless, other authors have shown contradictory results on this topic [84,85] and emphasized the need for further studies in pediatric patients.

We include the synthesis of the above-mentioned information in Table 3.

### 4.3. Other Aspects of the Gut Microbiome and Constipation—‘Show Must Go on…’

Several years after the above-mentioned study by Zoppi, a similar study indicated that children with constipation harbor a greater number of certain families and genera of the *Firmicutes phylum*, as well as a decrease in *Bacteroides* spp. [23]. Meji et al. assessed the gut microbiota composition of children with constipation as compared to healthy controls in terms of several species of major phyla, including *Actinobacteria, Firmicutes, Fusobacteria, Bacteroidetes, Verrumicobia,* and *Proteobacteria* [6]. The study revealed a decreased abundance of *Ruminococcus* spp. in children with functional constipation when compared to their matched controls [6]. Another recent similar study including children with functional constipation indicated that defecation frequency was negatively correlated with the abundance of *Clostridiales*, comprising genera like *Megasphaera*, *Oscilospira*, and *Ruminococcus* [78]. Moreover, fecal microbiota transplantation was also recently assessed as an innovative treatment combined with conventional treatment in children with slow-transit constipation, and it increased the cure rate by 30% when compared with conventional treatment [86].

Although *Bifidobacterium* and *Lactobacillus* strains remain the most commonly used probiotics, *Saccharomyces boulardii*, certain strains of *E. coli*, and a *Clostridium butyricum* strain are also approved to be used in the European Union [87], but further studies are needed for a clear recommendation in children with constipation.

## 5. Prebiotics, Postbiotics, Synbiotics, and Constipation in Children

Prebiotics are selectively fermented ingredients that have the ability to modify the contents and/or function of the gut microbiota, showing beneficial impacts on the host’s health [88]. Recently, the International Scientific Association for Probiotics and Prebiotics defined postbiotics as inanimate microorganisms and/or their components with positive effects on the host’s wellbeing [89]. Synbiotics are defined as the presence of both probiotics and prebiotics in the same product, and they are classified into two subtypes: synergistic, i.e., when the substrate is chosen for the selective growth of the co-administered probiotic(s); and complementary, i.e., the combination of a probiotic and a prebiotic designed to enhance the development of the autochthonous microflora [90].

Prebiotics such as fibers, fructo- or galactooligosaccharides, and polydextrose are commonly assessed along with a probiotic, i.e., as a synbiotic in the management of children with constipation. Thus, a study involving the aforementioned prebiotics and *L. casei, L. rhamnosus, L. plantarum*, and *B. lactis* as prebiotics proved that this synbiotic has a beneficial effect in children with constipation by increasing the weekly defecation frequency, and by improving bothersome symptoms like painful defecation and abdominal pain [91]. Another double-blind, randomized, placebo-controlled study involved 102 children with functional constipation, who were divided into three groups: group A—treatment consisted of liquid paraffin and placebo; group B—children were administered synbiotics (probiotics—*L. casei*, *L. rhamnosus*, *S. thermophilus*, *B. breve, L. acidophilus*, and *B. infantis*; prebiotic—fructooligosaccharide); and group C—treatment consisted of liquid paraffin and synbiotic. This study proved that although the defecation frequency increased in all three groups, it was higher in group C. Moreover, the authors also underlined that all children included in this study experienced improvements in fecal incontinence, abdominal pain, stool consistency, and painful defecation [92]. The effect of synbiotics also seems to depend on the patient’s age, according to the study of Mugambi et al., who proved that the supplementation of infant formula with synbiotics lacked any influence on stool consistency, crying, or colic [93]. Nevertheless, the authors noted that the supplementation of infant formula with prebiotics was associated with increased stool frequency, but it had no effect on stool consistency or crying. In terms of subtypes, the effects of complementary and synergistic synbiotics are similar, but Gomez Quintero et al. concluded that synergistic synbiotics might be considered as targeted therapy due to their potential ability to change the status of non-responders into responders [94].

Postbiotics represent an alternative to probiotics, with higher safety and stability due to their resistance to antibiotics, defined chemical structure, and ease of storage [95,96]. Unfortunately, the evidence regarding the role of postbiotics in the management of constipation is scarce. The available studies indicate that heat-killed *B. longum* CLA8013 improved stool volume and defecation-associated parameters. The effects of *Bifidobacterium longum* CLA8013 on bowel movement improvement were studied in a placebo-controlled, randomized, double-blind study [97]. *Lacticaseibacillus paracasei* metabolites enhanced the functioning of the intestinal barrier, improving the metabolism of water and sodium in mice with constipation [98], while *B. bifidum* MIMBb75 ameliorated abdominal pain in patients with irritable bowel syndrome [99]. One of the most recent studies on this topic assessed the impact of Probio-Eco, a postbiotic, fermented by B. lactis V9, *Lacticaseibacillus paracasei* Zhang, and *Lactiplantibacillus plantarum P-8,* using as sodium citrate, soy protein, and skimmed milk powder as substrates, in the management of adults with constipation, proving that it improves symptoms and stool straining, but it also induced beneficial changes in the gut microbiome, promoting anti-inflammatory responses [100]. Most of these effects seem to be related to its contents of bioactive compounds like organic and short-chain fatty acids, but also bacteriocins, which also reduce inflammation and suppress pathogenic bacteria [101,102]. In pediatric populations, postbiotics have so far been evaluated only in infants as a component of formulae, and the studies prove that postbiotics from *S. thermophilus* and *B. breve* C50 are well tolerated by both term and preterm infants, improving gastrointestinal symptoms, enhancing normal growth, and lowering inflammatory markers like calprotectin and tumor necrosis factor alpha [103,104,105,106,107]. Other studies performed in pediatric populations suggest that postbiotics might have benefic effects in children with diarrhea, atopic eczema, cow’s milk allergy, allergic rhinitis, and lactose malabsorption [108].

## 6. Fecal Microbiota Transplantation in Children with Constipation

Fecal microbiota transplantation (FMT) represents the administration of fecal material originating from a healthy donor’s distal intestinal microbiota to an individual who presumably has an alteration in their gut microbial composition, or a condition associated with dysbiosis [109]. Currently, the only clear indication for FMT in children is *Clostridium difficile* infection under the following conditions: recurrence or relapse with a minimum of three moderate or severe episodes with no response to 6–8 weeks of vancomycin taper, or a minimum of two episodes of severe infection requiring admission and accompanied by significant morbidity; moderate forms of infection refractory to standard therapy after at least 1 week; and severe forms of infection showing no response after 48 h of standard therapy [110]. Nevertheless, FMT might be associated with severe adverse events even in this group of patients (ART). In terms of functional constipation, FMT has so far been performed only in adult populations, but in small samples showing promising results, with an up to 30% higher cure rate than conventional treatment [86,111]. A more recent study underlined that FMT improves not only constipation parameters in adults but also depression and anxiety [112].

## 7. Future Directions and Study Limitations

The topic of microbiota, as well as pre-, pro-, syn-, and postbiotics, in the management of pediatric functional constipation remains a controversial topic that deserves special attention in further studies, since functional constipation is one of the most common problems in children, and although it usually responds to pharmacological treatment, the long-term prognosis is poor due to frequent relapses after treatment. Thus, future research should focus more on precisely characterizing the peculiarities of gut microbial content in children with this condition. This could be the first step for designing a targeted therapy based on compounds such as biotics that might precisely repair the ‘defects’, resulting in short- and long-term favorable evolution.

Although the role of probiotics in the management of children with constipation is relatively thoroughly investigated, that of the other biotics is far from being elucidated. Postbiotics might be a promising targeted therapy for alleviating all symptoms related to this condition, but future clinical trials involving pediatric patients should be performed in order to clearly establish their role. In addition, synbiotics might represent an even better alternative as compared to probiotics, due to their ability to enhance the proper development of the resident microbiota and to promote the activity of the probiotic in its composition.

FMT should be considered a rescue therapy and used only for particular cases who are refractory to conventional treatment or whose long-term evolution is not favorable despite multiple treatments. Moreover, it should be performed in specialized centers in order to preempt potential adverse events, or to treat them promptly if they occur. Thus, centers that have the ability to perform FMT in children with constipation should be aware of the importance of publishing their experience.

The major limitations of most of the studies involving pediatric patients with constipation is the lack of a holistic approach to these children, consisting of the combined assessment of diet, lifestyle, and life events that might have triggered the onset of constipation. Moreover, almost all studies have failed in characterizing the host’s gut microbiome before initiating the treatment. A potential limitation of our review is that we did not also assess the impact of microbiota and -biotics in children with secondary constipation, but since this is a large topic, we aim to target it in future research.

## 8. Conclusions

The supplementation of probiotics in children with constipation is definitely a challenging and study-worthy topic today. Although several studies clearly indicate their beneficial effects in ameliorating symptoms of constipation, especially regarding bowel movement frequency, the heterogeneity of the current studies and the relative scarcity of clinical trials involving pediatric subjects so far hinder their current use as a standard in the management of children with constipation, as a consequence of physicians’ reluctance. Nevertheless, according to most of the aforementioned data, probiotics might be a reliable, safe, and effective option for treating symptoms of constipation in pediatric patients.

## Figures and Tables

**Table 1:** The impact of microbiota on gut motility.

Reference (Author, Year)	Mechanisms	Effects
Kwiatkowska et al., 2021 [5]	Changes in pH in the intestine Regulation of butyric acid (BA) concentration Production of methane in the intestinal lumen Neuroendocrine factors Bile acid metabolism	Abnormal pH prevents the development of *Lactobacillus* and *Bifidobacterium*. Facilitates smooth muscle contraction in the colon. Excessive BA inhibits mucin secretion, decreased stool volume, decrease of *Prevotella* species Intestinal distension, smooth muscle contractility ↓, slowing peristalsis. *Lactococcus*, *Streptococcus*, *Escherichia*, and *Candida* increase the concentration of serotonins, *Lactobacillus* and *Bifidobacterium* increase gamma-aminobutyric acid, and *Bacillus* spp., *Escherichia* spp*.,* and *Saccharomyces* spp. increase norepinephrine. Deconjugation and dehydroxylation of primary bile acids.
Simrén et al., 2013 [19]	Abnormal microbiota activates mucosal innate immune responses, consequently increasing epithelial permeability, dysregulating the enteric nervous system, and activating nociceptive sensory pathways Gastric acid secretion, fluid, anticommensal sIgA, antimicrobial peptide production, and gastrointestinal (GI) motility affect gut microbiota composition Antibiotics, dietary modifications	Increased abundance of *Bacteroidetes* and *Enterobacteriaceae.*
Khalif et al., 2009 [20]	Changes in systemic immunity, the fecal flora, and intestinal permeability	Decreased *Bifidobacterium* and *Lactobacillus.* Higher serum ovalbumin concentrations. Elevated titers of antibodies to *Escherichia coli* and *S. aureus.*
Zhao et al., 2016 [21]	Short-chain fatty acids (SCFAs) stimulate ileal propulsive contractions	Increased peristalsis. Decrease in obligate bacteria (e.g., Lactobacillus, Bifidobacterium, and Bacteroides spp.) and increase in potentially pathogenic microorganisms (e.g., *Pseudomonas aeruginosa* and *Campylobacter jejuni*). Increase in butyrate-producing genera (*Coprococcus*, *Roseburia*, *Faecalibacterium*).
Khan et al., 2018 [15]	Bacterial endotoxin lipopolysaccharide synthetized by Gram-negative bacteria (e.g., *Enterobacteria*)	Delay in gastric emptying and sphincter dysfunction, leading to decreased bowel movements.
Husebye et al., 2001 [29]	*Bifidobacteria* and *Lactobacillus* spp. augment the release of serotonin *Micrococcus luteus* and *Escherichia coli* strains	Pro-motility effects by reducing the migrating myoelectric complex period and subsequent acceleration of small gut transit. Inhibitory effect on bowel transit.
Wong et al., 2006 [30]	Short-chain fatty acids (SCFAs), including acetate, butyrate, and propionate, produced in anaerobic conditions during fermentation.	Butyrate nourishing the colonic mucosa, promoting cell differentiation, cell-cycle arrest, and apoptosis of transformed colonocytes.
Barbara et al., 2005 [31]	*Clostridia, Bifidobacteria*, and *Faecalibacterium prausnitzii* stimulate the enteric cholinergic reflex	Stimulating the release of GLP-1 or polypeptide YY from gut mucosal cells.
Jennings et al., 2009 [32]	SCFAs—serotonin release from enterochromaffin cells via vagal sensory fibers’ promotion	Acceleration of bowel transit.
Canani et al., 2011 [35]	Butyrate	Increased concentrations—increasing bowel transit. Normal concentrations—inducing constipation due to colonic electrolyte and water absorption and lowering colonic mucin secretion.
Begley et al., 2006 [36]	Gut microbes secreting enzymes that hydrolyze bile acids into non-reabsorbable secondary bile acids	Binding to receptors and forming the G-protein-coupled bile acid receptor, and enabling enterochromaffin cells and intrinsic primary afferent neurons to release serotonin- and calcitonin-generated peptides, acting as a trigger for the bowel’s peristaltic reflex.

**Table 2:** The role of *Bifidobacterium* spp. in children with constipation.

Reference (Author, Year)	Type of Study	Study Population	Objectives	Prebiotic, Probiotic, Synbiotic	Conclusions/Outcomes
Kitajima et al., 1997 [48]	Prospective randomized clinical study	VLBW Age: preterm infants	Colonization of the bowels with *Bifidobacterium breve YIT4010*	*Bifidobacterium breve YIT4010*, 0.5 × 10^9^ CFU, 28 days	Less gas accumulation within the stomach, improved weight gain, no side effects
Tabbers et al., 2011 [43]	Non-randomized, non-placebo-controlled pilot study	n = 20 Age: 3 to 16 years FC	Evaluation of defecation frequency and stool consistency	*Bifidobacterium breve* 10^8^–10^10^ CFU, 4 weeks	Increasing stool frequency, positive effect on stool consistency, decreasing the number of fecal incontinence episodes, diminishing abdominal pain
Zoppi et al., 1998 [54]	Randomized, double-blind, placebo-controlled	n = 42 Age: 5–14 years FC	Evaluation of composition of the intestinal ecosystem in chronic FC; calcium polycarbophil effect on constipation	Calcium polycarbophil (0.62 g/twice daily) or placebo	Greater abundance of the genera *Bifidobacteria* and *Clostridia* No generalized overgrowth
Moro et al., 2002 [55]	Randomized, placebo-controlled trial	n = 90 Age: term infants	Day 1 and day 28, the fecal species, colony-forming units (cfu), and pH were measured, along with stool characteristics, growth, and side effects	0.4 g/dL or 0.8 g/dL oligosaccharides added to test formulae	Mixture of galacto- and fructooligosaccharides stimulating growth of *Bifidobacteria* and *Lactobacilli* Softer stool
Bongers et al., 2007 [56]	Double-blind, randomized, crossover trial	n = 38 Age: 3–20 weeks FC	Effect of high concentration of *sn-2* palmitic acid, a mixture of prebiotic oligosaccharides, and partially hydrolyzed whey protein on stool characteristics	*sn-2* palmitic acid, prebiotic oligosaccharides, and partially hydrolyzed whey protein	Softer stools No effect on defecation frequency
McCarthy et al., 2003 [57]	Double-blind, placebo-controlled trial	Interleukin-10-knockout mice	Effects of *Lactobacillus salivarius subspecies salivarius 433118* and *Bifidobacterium infantis 35624* against placebo on enterocolitis and the intestinal microflora; compare the systemic immunological response	*Lactobacillus salivarius 433118*, 10(9) CFU/mL *Bifidobacterium infantis 35624,* 10(8) CFU/mL and unmodified milk	Significantly attenuated colitis Reduced pro-inflammatory cytokine production
Makizaki et al., 2019 [58]	Crossover control trial	Six-week-old male Sprague Dawley rats	Effectiveness of BBG9-1 in constipation and its influence on intestinal flora using a constipation model that reflects constipation caused by insufficient dietary fiber	Low-fiber diet and *Bifidobacterium bifidum G9-1* for 14 days	Increased defecation frequency Increased fecal weight Alleviation of dysbiosis Increase in butyric acid
Guerra et al., 2011 [59]	Crossover, double-blind, controlled trial	n = 59 Age: 5–15 years FC	Effect of goat yogurt with *Bifidobacterium longum* on FC	10^9^ CFU/mL *Bifidobacterium longum (B. longum)* containing yogurt	Improved defecation frequency, alleviated defecation and abdominal pain
Astó et al., 2022 [60]	Observational pilot trial	n = 36 Age: 1–10 month FC and/or infant colic (IC)	Probiotic potential of *Bifidobacterium longum KABP042* and *Pediococcus pentosaceus KABP041* in FC and IC	*Bifidobacterium longum KABP042* and *Pediococcus pentosaceus KABP041*	Improved symptoms Positive anti-infectious effect by antagonizing enteropathogens
Chen et al., 2021 [62]	Randomized, double-blind, placebo-controlled trial	n = 112 Age < 2 months IC	Efficacy of *Bifidobacterium longum* CECT7894 (KABP042) and *Pediococcus pentosaceus* CECT8330 (KABP041) in IC	*Bifidobacterium longum* CECT7894 and *Pediococcus pentosaceus* CECT8330 (1 × 10^9^ colony-forming units)	Shorter crying time and decreased crying episodes Improving fecal consistency
Chen et al., 2024 [63]	Randomized, double-blinded, placebo-controlled	n = 131 Age: 0–6 years FC	Efficacy and safety of *Bifidobacterium animalis subsp. lactis XLTG11*	*Bifidobacterium animalis subsp. lactis XLTG11* 1 × 10^10^ CFU/sachet	Increase in defecation frequency Upregulating short-chain fatty acid-related genes Downregulating those related to methane metabolism
Bekkali et al., 2007 [64]	Observational pilot study	n = 20 Age: 4–16 years FC	Effects of *Bifidobacterium infantis*, *Bifidobacterium longum*, *Bifidobacterium bifidum*, *Lactobacillus plantarum*, *Lactobacillus rhamnosus*, and *Lactobacillus casei* on frequency of bowel movements (BMs), stool consistency, and number of fecal incontinence episodes	Mix of *Bifidobacterium infantis*, *Bifidobacterium longum*, *Bifidobacterium bifidum*, *Lactobacillus plantarum*, *Lactobacillus rhamnosus*, and *Lactobacillus casei*, 4 × 10^9^ CFU, 4 weeks	Increased defecation frequency Fecal consistency improvement Reduction in fecal incontinence episodes

Legend: FC—functional constipation, IC—infant colic, CFU—colony-forming unit.

**Table 3:** The role of *Lactobacillus spp.* in children with constipation.

Reference (Author, Year)	Type of Study	Study Population	Objectives	Prebiotic, Probiotic, Synbiotic	Conclusions/Outcomes
Jomehzadeh et al. 2020 [73]	Case–control study	n = 40 FC n = 40 healthy volunteers Age: 4–18 years	To compare *Lactobacillus* species in FC vs. healthy volunteers with quantitative real-time polymerase chain reaction (qPCR)	*Lactobacillus* strains	Decrease in *Lactobacillus reuteri* and *Lactobacillus fermentum* in FC
Jomehzadeh et al. 2022 [68]	Case–control study	n = 40 FC n = 40 healthy volunteers (HV) Age: 4–18 years	To compare the prevalence and quantity of *Lactobacillus* species in FC vs. healthy volunteers with species-specific PCR and qPCR	*Lactobacillus* strains	No significant differences in the prevalence of *Lactobacillus* species in HV and FC. *L. paracasei* and *L. planetarium* species were predominant FC had a smaller quantity of total lactobacilli per milligram of stool The presence of *Lactobacilli* in the gut is insufficient for preventing functional constipation Quantity is essential for fulfilling their function
Mikelsaar et al. 2002 [71]	Parallel-group trial	n = 71 Estonian n = 65 Swedish Age: 1–2 years	To compare the predominant *Lactobacilli* in Estonian and Swedish, children with a low and high prevalence of allergy	*Lactobacillus* strains	Fermentation types were similar *L. plantarum* strains were encountered only from Estonian children Region-specific differences in colonization with particular *Lactobacilli*
Contreras et al. 2020 [77]	Double-blind, randomized, placebo-controlled clinical trial	n = 37 cerebral palsy and constipation Age: 14 to 60 months	Efficacy of a probiotic (*Lactobacillus reuteri* DSM 17938), a prebiotic (agave inulin), and a synbiotic on the stool characteristics in children with cerebral palsy and chronic constipation.	*L. reuteri* DSM 17938 plus placebo Inulin plus placebo *L. reuteri* DSM 17938 plus agave inulin Two placebos 28 days	Stool pH decreased in the probiotic group Prebiotic group: improvement in stool consistency *L. reuteri* and/or agave: excessive stool retention and painful defecation episodes decreased
Kubota et al. 2020 [78]	Prospective, double-blind, placebo-controlled, randomized, and parallel-group trial	n = 60, FC Age: 6 months–6 years	To evaluate the efficacy of the probiotic *L. reuteri* DSM 17938 and the laxative magnesium oxide (MgO) in chronic FC	*L. reuteri* DSM 17938 plus placebo *L reuteri* DSM 17938 plus MgO	*L. reuteri* DSM 17938 and MgO present positive effects on defecation frequency MgO was associated with decreased stool consistency and reduction in the genus *Dialister*
Coccorullo et al. 2010 [79]	Double-blind, randomized, placebo-controlled trial	n = 44, FC Age: 6–11 months	To evaluate the beneficial effects of *Lactobacillus reuteri* (DSM 17938) in infants with FC (frequency of bowel movements per week, stool consistency, presence of inconsolable crying episodes)	Group A: probiotic *L. reuteri* (DSM 17938) Group B: placebo	*L. reuteri* improved bowel movements and defecation frequency
Indrio et al. 2014 [81]	Prospective, multicenter, double-masked, placebo-controlled, randomized clinical trial	n = 589 Age: < 1 week, term infants	*Lactobacillus reuteri DSM 17938* to reduce the onset of colic, gastroesophageal reflux, and constipation	*Lactobacillus* reuteri DSM 17938, three months	Prophylactic use of *L. reuteri* DSM 17938 reduced the crying time and the regurgitations per day, and increased the number of evacuations

Legend: FC—functional constipation, HV—healthy volunteers.

## Data Availability

No new data were created or analyzed in this study. Data sharing is not applicable to this article.

## Abbreviations

The following abbreviations are used in this manuscript:

FC	Functional constipation
SCFA	Short-chain fatty acid
